# Response to the Comment on “A Systematic Review and Meta-Analysis of Hospitalised Current Smokers and COVID-19”

**DOI:** 10.3390/ijerph17249574

**Published:** 2020-12-21

**Authors:** Jesus González-Rubio, Carmen Navarro-López, Elena López-Najera, Ana López-Najera, Lydia Jiménez-Díaz, Juan D. Navarro-López, Alberto Najera

**Affiliations:** 1 School of Medicine, Centre for Biomedical Research, University of Castilla-La Mancha, 02008 Albacete, Spain; jesus.Gonzalez@uclm.es; 2 Hospital General La Mancha Centro, Servicio de Salud de Castilla-La Mancha, Alcazar de San Juan, 13600 Ciudad Real, Spain; mdelnl@sescam.jccm.es; 3 Gerencia de Atención Primaria, Salud de Castilla y Leon, 05003 Ávila, Spain; malopezna@saludcastillayleon.es; 4 Gerencia de Emergencias Sanitarias, Salud de Castilla y Leon, 47407 Salamanca, Spain; amlopezn@saludcastillayleon.es; 5 School of Medicine, Centre for Biomedical Research, University of Castilla-La Mancha, 13071 Ciudad Real, Spain

This is a reply to the comment by Ivan Berlin and Daniel Thomas on our recently published work [1]. The authors of “A Systematic Review and Meta-Analysis of Hospitalised Current Smokers and COVID-19” greatly appreciate Ivan Berlin and Daniel Thomas’ comments. However, we believe that the comment, as a whole, presents a biased interpretation of the information.

Berlin and Thomas start their comment stating that results “confirmed the protective effect of current smoking on the likelihood of hospitalization”. However, in our opinion, they make an incorrect interpretation of the phrase (it is taken out of context), since such sentence was used in the *L’Abbé* graphic. The so-called [2] graphs are a good way to visualize data based on event rates. In these graphs the event rate in the intervention group of a study is plotted against the event rate in the control group. Then, if a type of intervention to have a protective effect (i.e., to make an adverse outcome such as less likely hospitalization) is expected, the studies should mainly be located in the lower right corner of the *L’Abbé* graph. This is the case in our paper: the “protective effect” is the fact of being a current smoker over the fact of hospitalization. It is no stated in any case that the protective effect is tobacco, but rather that there is a lower prevalence of smoking among patients hospitalized for COVID-19 than expected.

It is likely that this association does not involve a causal relationship and, of course, as it is clearly indicated in the paper, smoking is not recommended as prevention or treatment of COVID-19. The association could be due to many factors that could include common smoking habits, increased likelihood of cross-immunity when smokers are infected by other coronavirus [3], anti-inflammatory effect of nicotine [4,5,6], increased hygienic habits, giving up smoking when became sick being considered non-smokers, the definition of “current smoker” is not always provided, etc. A detailed argumentation of all these potential issues is included in the discussion section of the paper. Coming back to the main focus of the results, what seems clear is the finding of an underrepresentation of smokers among COVID-19 hospitalized patients, which has been confirmed by other meta-analysis [7] and even by the European Commission [8].

Berlin and Thomas also stated that “none of 20 the included papers reported on biochemical verification of smoking status”. We agree with this assessment and consider that it would be very interesting to include it (biochemical verification and COVID-19) in future reviews when these data are available, especially when most recent studies indicate a negative association between smoking prevalence and occurrence of COVID-19 at the population level in 38 European countries [9].

Furthermore, Ivan Berlin and Daniel Thomas indicated that “It is not surprising that the prevalence rate of smoking is substantially lower among individuals hospitalized for COVID-19 in each country than in their general population and shows a relatively low measure of dispersion”. Contrariwise, we do find it surprising that the prevalence of smoking is substantially lower among people hospitalized by COVID-19, considering that smoking is known to increase the onset of multiple respiratory diseases [10]. In addition, smokers are known to have a significantly higher risk of chronic respiratory disease and acute respiratory infections [11]. Even current smokers have an increased risk of developing *influenza* compared to non-smokers [12]. Smoking was also significantly associated with MERS-CoV [13].

Finally, Berlin and Thomas stated that “speculation about biological mechanisms is adequate only if a cause to effect relationship between smoking and hospitalization for COVID-19 is demonstrated in prospective studies. A protective effect should be evoked with extreme caution because a biased message may induce smokers to continue to smoke or to relapse to smoking to protect themselves from COVID-19”. We are sorry that the authors have greatly misrepresented our conclusion that in no case was what they described. However, the fact that tobacco is a poison that kills eight million people a year in the world does not mean that an epidemiological finding, which might open new research lines, should be despised. Our analysis is based on consistent epidemiological data and proposes a physiological substrate already demonstrated for inflammatory diseases [6,14]. This is the advantage of working in a multidisciplinary team like ours formed by doctors, epidemiologists and physiologists. We believe that the pandemic has been the domain of epidemiologists, virologists, behavioral psychologists and respiratory researchers, and the neuro sides to it are just coming through [15].

In our opinion, Berlin and Thomas have obviated these multidisciplinary approaches in their comment, and have distorted the message of our study, placing the focus on an erroneous interpretation, suggesting that our findings might induce smokers to keep or go back to smoking to protect themselves from COVID-19. In our work, the surprising finding is just the opposite: that the cases of COVID-19 do not group together with smokers. The clue must be investigated.
